# Transcriptome-Wide Identification and Functional Characterization of CIPK Gene Family Members in *Actinidia valvata* under Salt Stress

**DOI:** 10.3390/ijms24010805

**Published:** 2023-01-02

**Authors:** Shichao Gu, Muhammad Abid, Danfeng Bai, Chen Chen, Leiming Sun, Xiujuan Qi, Yunpeng Zhong, Jinbao Fang

**Affiliations:** Key Laboratory for Fruit Tree Growth, Development and Quality Control, Zhengzhou Fruit Research Institute, Chinese Academy of Agricultural Sciences, Zhengzhou 450009, China

**Keywords:** kiwifruit, salt tolerance, CIPK, ion homeostasis

## Abstract

Fruit plants are severely constrained by salt stress in the soil due to their sessile nature. Ca^2+^ sensors, which are known as CBL-interacting protein kinases (CIPKs), transmit abiotic stress signals to plants. Therefore, it is imperative to investigate the molecular regulatory role of CIPKs underlying salt stress tolerance in kiwifruit. In the current study, we have identified 42 *CIPK* genes from *Actinidia. valvata* (*A.valvata*). All the *AvCIPK*s were divided into four different phylogenetic groups. Moreover, these genes showed different conserved motifs. The expression pattern analysis showed that *AvCIPK11* was specifically highly expressed under salt stress. The overexpression of *AvCIPK11* in ‘Hongyang’ (a salt sensitive commercial cultivar from *Actinidia chinensis*) enhanced salt tolerance by maintaining K^+^/Na^+^ homeostasis in the leaf and positively improving the activity of POD. In addition, the salt-related genes *AcCBL1* and *AcNHX1* had higher expression in overexpression lines. Collectively, our study suggested that *AvCIPK11* is involved in the positive regulation of salt tolerance in kiwifruit.

## 1. Introduction

Abiotic stresses are a global constraint to agricultural development. Among them, salt stress is a major factor that limits land utilization and food output [1]. It inhibits seed germination, plant growth, development, and fruit yield in fruit trees. The salt tolerance ability in plants varies depending on their internal salt tolerance mechanisms. Salt stress causes damage to plants by imposing osmotic stress, ionic stress, and oxidative stress [2]. The overaccumulation of ions, i.e., Na^+^, K^+^, Ca^2+^, Mg^2+^, Cl^−^, SO_4_^2−^, and NO_3_^−^, significantly inhibits plant growth and development [3]. Additionally, toxic ions are more likely to accumulate in older leaves, resulting in leaf damage and death. Therefore, it is highly prudent to unravel the molecular mechanism of salt tolerance in fruit plants.

The influx of inorganic ions in plant cells plays an important role in the regulation of salt tolerance in fruit trees. Within plant cells, Ca^2+^ is an important nutrient and the second messenger within plants that regulate ion homeostasis, the stress response (abiotic and biotic), and stomatal opening [4]. The overaccumulation of Ca^2+^ within plants under salt stress enhances signal transduction to protect it from fatal damage [5,6]. The CIPK gene family plays an important role in plant responses to abiotic stresses, including salt, drought, flooding, high temperatures, and low temperatures [7,8]. CIPK family members harbor the SNF domain at the N-terminal and the FISL/NAF domain at the C-terminal [9]. In *Arabidopsis*, a salt overly sensitive (SOS) pathway containing SOS3 ((a calcineurin B-like protein-CBL) [10], SOS2 (a CIPK protein) [11], and SOS1 (a Na^+^/H^+^ transporter activated by SOS2) [12] maintains cellular homeostasis under salt stress by transporting calcium ion signals. SOS3 can interact with SOS2 via the FISL motif allowing SOS2 to transition from the self-inhibited state to the kinase state in a calcium-dependent manner [12]. Excess Na^+^ under salt stress can be transported out of the cell through the SOS pathway via the plasma membrane Na^+^/H^+^ antiporter SOS1. The CBL4-CIPK14 (also known as SOS3-SOS2) is the earliest reported CBL-CIPK module in *Arabidopsis* that plays a positive regulatory role in salt stress tolerance by activating the plasma membrane (PM)-localized Na^+^/H^+^ antiporter SOS1 [13]. Similarly, the AtCBL4-AtCIPK24 module enhanced salt tolerance in *Arabidopsis* roots by activating the Na^+^/H^+^ antiporter AtNHX7 (SOS1) and vesicular H^+^-ATPase [14]. The CBL1/9-CIPK23 complex stimulated the potassium transporter proteins HAK5 and AKT1 to enhance the potassium absorption by the plant under low potassium conditions [15,16]. Additionally, in a low potassium state, CBL1 and CBL9 transport CIPK23 to the plasma membrane, where it can phosphorylate and activate AKT1 or HAK5 to increase K^+^ entry into the cell [17,18,19]. In addition, in a low-potassium environment, potassium channels in the vesicle membrane can also be activated by CBL-CIPK thus maintaining ionic homeostasis [20]. The CBL2-CIPK11/14 was involved in the regulation of the H^+^-ATPase, which in turn controlled the PH of the cells in *Arabidopsis* [21]. Additionally, CIPK genes were reported to regulate the transport of phytohormones in tobacco to improve salt tolerance [22]. The overexpression of *BrCIPK1* improves plant abiotic stress tolerance by promoting proline synthesis in rice [23]. The CIPKs improved the stress tolerance of plants by modulating the activities of reactive oxygen species (ROS) scavengers (POD, SOD, and CAT), and reducing H_2_O_2_ and MDA content [24,25].

The CIPK gene family has been identified in numerous plants. There were 25 CIPK members in the *Arabidopsis* [26]. In total, 23 CIPK genes in *Brassica napus* L. [27], 30 CIPK genes in *Oryza sativa* [28], 43 CIPK genes in *Zea mays* L. [29], 27 CIPK genes in *Populus* [30], 20 CIPK genes in *Vitis vinifera* [31], and 34 CIPK genes *Malus Domestica* [32], as well as 80 and 78 CIPK genes in *Gossypium hirsutum* and *G. barbadense*, respectively [33], and 27 CIPK genes in *Solanum tuberosum* [34]. The molecular regulatory mechanism of CIPKs under salt stress is unclear and demands more exploration.

The kiwifruit (*Actinidia chinensis* Planch.) is a perennial deciduous fruit tree belonging to the genus *Actinidia* L. It is rich in nutrition and has various health functions. Previously, we have obtained promising results for a superior quality rootstock material ZMH through screening kiwifruit germplasm resources against salt stress [35,36]. In the current study, we comprehensively analyzed the structure and function of CIPK gene family members in the ZMH transcriptome [37]. Our findings will improve the understanding of calcium signaling in salt stress. Moreover, it will help researchers to develop salt tolerant cultivars to tackle the damaging effects of climate change on the kiwifruit industry.

## 2. Results

### 2.1. Identification and Phylogenetic Analysis of AvCIPK Genes in A. valvata

**Figure 1:**
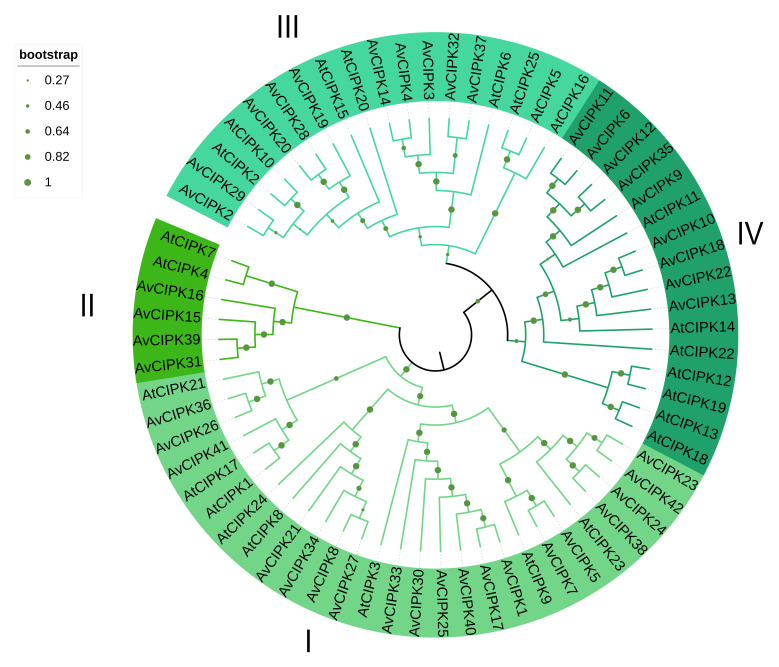
The phylogenetic analysis of the AtCIPK and AvCIPK proteins. Each clade in the phylogenetic tree is represented by a different color. I–IV represent the different groups.

We used the prediction tools, InterProscan and ScanProsite, to identify 42 AvCIPK proteins containing the conserved domain. The 42 AvCIPK family members were renamed as AvCIPK1-42. Proteins encoded by the AvCIPK genes varied in length from 412 to 477aa (Table S1). The relative molecular weights of CIPK kinase proteins varied from 47.12–54.50 KDa. The predicted theoretical pI ranged from 6.85 to 9.35. The instability index was 25.8145.44 and the aliphatic index was 82.96–93.61 The hydropathicity of AvCIPK ranged from –0.408 to –0.151, which indicated all CIPK proteins were hydrophobic (Table S1). A phylogenetic tree was constructed for 42 AvCIPK and 25 AtCIPK proteins by using the neighbor-joining (NJ) method (Figure 1). The protein sequences of AvCIPKs are presented in Supplementary Table S2. Based on the phylogenetic tree, the AvCIPKs were classified into four distinct groups (I–IV). Group I contained the greatest number of AvCIPKs (19 proteins) and group II had the least number of AvCIPK proteins (four proteins). Groups III and IV contained 10 and 9 AvCIPK proteins, respectively.

### 2.2. Analysis of Conserved Motif and Gene Structure for AvCIPKs

**Figure 2:**
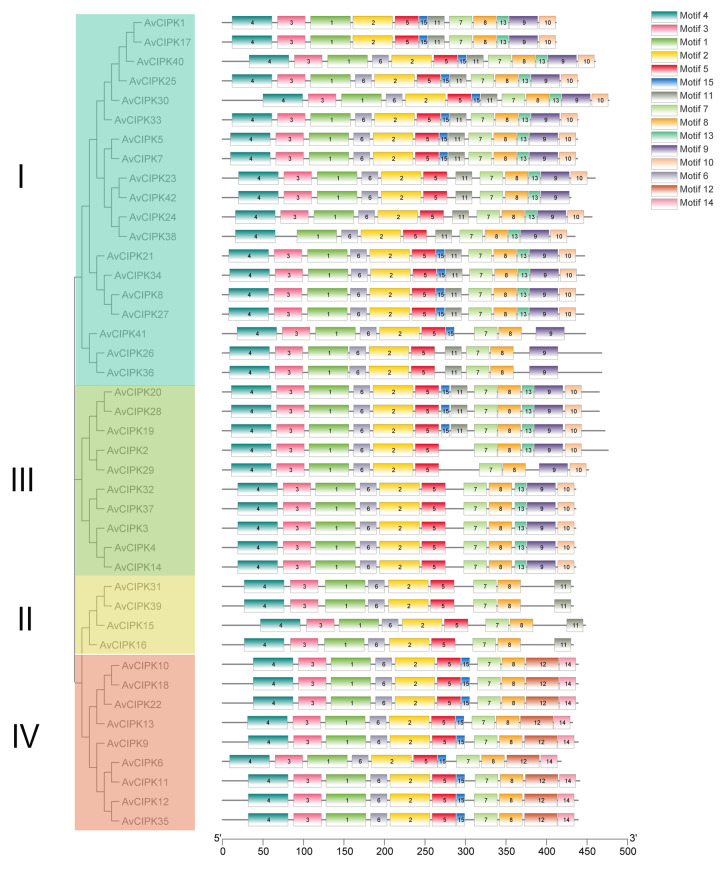
Analysis of phylogenetic relationships and conserved motifs of AvCIPK proteins. The phylogenetic groups and conserved motifs are presented in different colors. I–IV represent the different groups.

**Figure 3:**
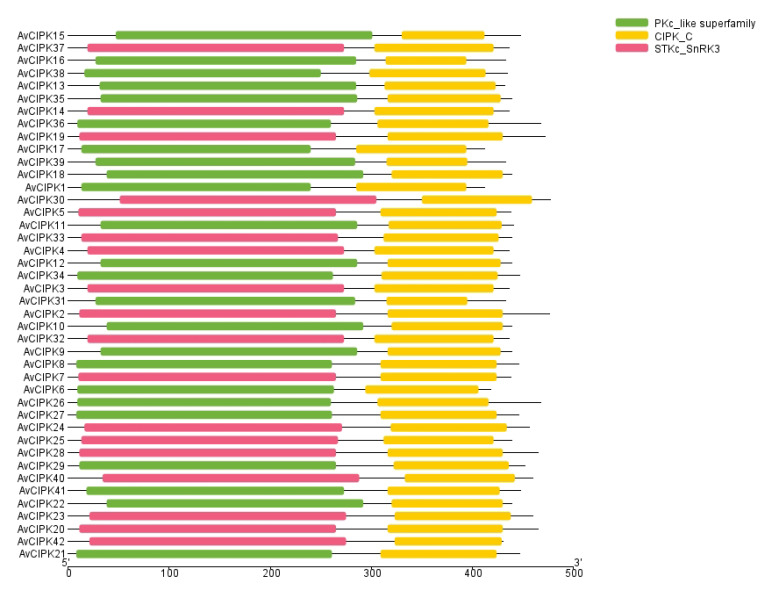
Identification of conserved domains in AvCIPK protein sequences.

We identified a total of 15 conserved motifs in 42 AvCIPK protein sequences (Figure 2). Interestingly, members of the same phylogenetic group had similar motif compositions. Motif 1, motif 2, motif 4, motif 5, motif 7, and motif 8 were identified in all AvCIPKs, indicating that these conserved motifs might play an important role in the function of AvCIPKs. Motif 3 was found in all AvCIPKs except for AvCIPK38. Similarly, motif 6 was contained by all AvCIPKs except for AvCIPK1 and AvCIPK17. Some motifs were specific to a distinct phylogenetic group, i.e., Motif 15 in groups I, III, and IV; motif 11 in groups I, II, and III; motifs 9, 10, and 13 in groups I and III; and motifs 12 and 14 in group IV. The amino acid sequence of the conserved motifs in AvCIPK protein shows that all its members have motif 7 and motif 8 (Figure S1). These two motifs are essential for the function of CIPK protein. Moreover, conserved domain analysis demonstrated that all 42 AvCIPKs contained a CIPK_C domain (Figure 3). The existence of the conserved domain ensures the stability of its function. 

### 2.3. Expression Profiles of AvCIPK Genes

**Figure 4:**
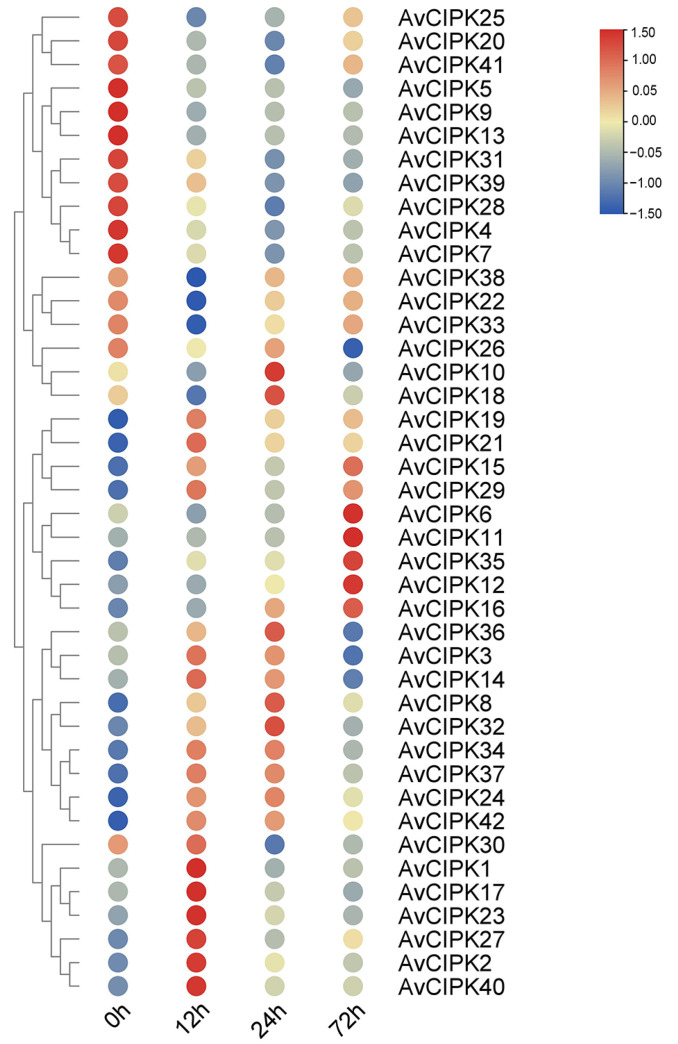
The heatmap for expression profile of kiwifruit CIPK family members under different time points of salt stress.

We investigated the expression patterns of 42 *AvCIPKs* in the salt-tolerant plant material ‘ZMH’ at 0 h, 12 h, 24 h, and 72 h after salt stress (Figure 4). The FPKM values were used to estimate the expression profile of *AvCIPKs* for screening the candidate genes associated with salt tolerance. The analysis revealed that *AvCIPK* genes had distinct expression profiles after salt stress. Some of the *AvCIPK* genes (*AvCIPK1, AvCIPK2, AvCIPK17, AvCIPK23, AvCIPK27,* and *AvCIPK40)* were expressed at the initial stages of salt stress (0 h and 12 h), while some *AvCIPK* genes (*AvCIPK6, AvCIPK11, AvCIPK12, AvCIPK16,* and *AvCIPK35)* were induced at the final stages (24 h and 72 h). We are interested in plant salt tolerance under salt stress. Therefore, we paid more attention to transcriptomic changes between 0h and 72h. It is well known that the CIPK genes played an important role in maintaining low Na^+^ levels in cells via the SOS3-SOS2-SOS1 module in plants [12]. The heatmap results identified *AvCIPK6, AvCIPK11, AvCIPK12, AvCIPK16*, and *AvCIPK35* as salt-responsive genes. Based on annotation results, we knocked out AvCIPK12 and AvCIPK35 (low quality protein) and AvCIPK16 (does not belong to the SOS3-interacting protein) (Table S3). The results showed that the expression of the *AvCIPK11* gene from the phylogenetic group IV increased significantly more than *AvCIPK6* after salt stress. Based on the above results, the AvCIPK11 gene was selected as the core salt-responsive gene for subsequent analysis.

### 2.4. Subcellular Localization Analysis of AvCIPK11

**Figure 5:**
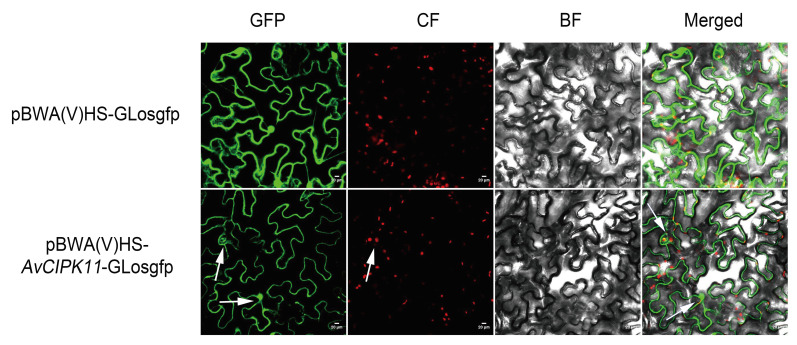
Subcellular localization analysis of the AvCIPK11 protein. GFP: Green fluorescence protein; CF: Chloroplast fluorescence; BF: Bright field; Merged: Merged field for GFP and CF. The scale bar was set at 20μm. Arrows represent where the protein is localized.

To investigate the subcellular localization of AvCIPK11, the pBWA(V)HS-AvCIPK11-GLosgfp vector was transiently expressed in *N. benthamiana* leaves by the *Agrobacterium*-mediated transformation method. An empty vector containing a GFP was considered as a negative control. The GFP fluorescence signals were detected under a confocal microscope (Figure 5). The cells containing the recombinant plasmid showed GFP fluorescence signals in the nucleus and cytoplasm. The result indicated that *AvCIPK11* might encode a protein localized in the nucleus and cytoplasm.

### 2.5. Overexpression of AvCIPK11 Enhances Salt Tolerance of A. chinensis

**Figure 6:**
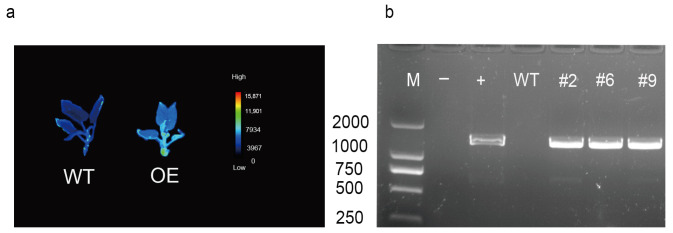
Identification of positive transgenic *A. chinensis* plants. (**a**) Based on the expression of GFP reporter genes in plants. (**b**) The PCR detection of T_1_ *A. chinensis* transgenic lines. M, 2000 bp marker; -, ddH2O; +, the recombinant plasmid containing *AvCIPK11*; WT, wild type kiwifruit; and #2, #6, #9, positive transgenic lines. The forward primer was designed at 5′of 35S and the reverse primer was designed at 3′of *AvCIPK11*. The pair of primers can be found in supplementary Table S4.

**Figure 7:**
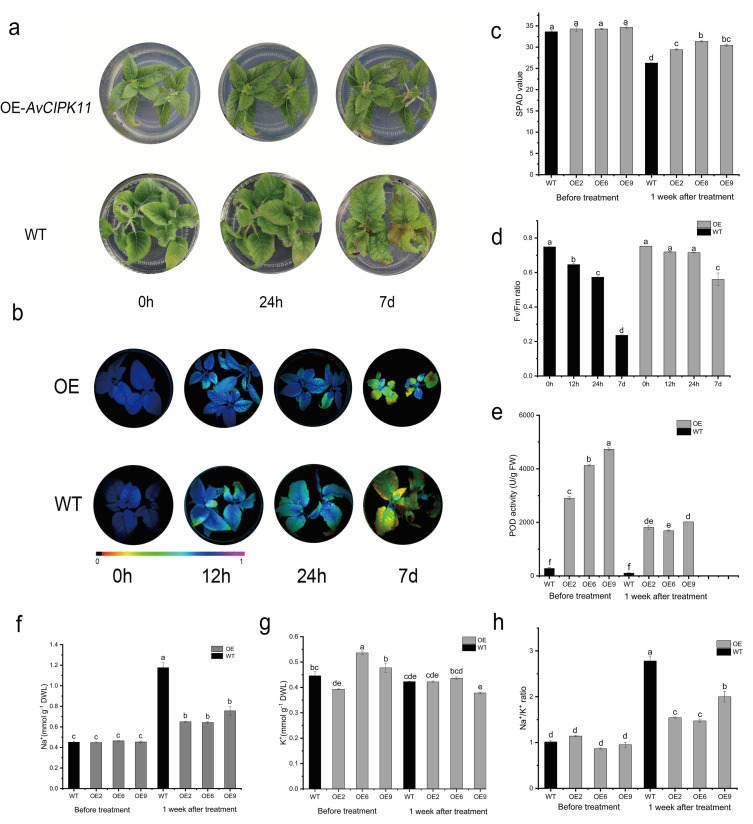
Functional validation of *AvCIPK11* in kiwifruit under salt stress. (**a**) Phenotypes of WT (*A. chinensis* cv. ‘Hongyang’) and OE kiwifruit plants under normal and salt stress conditions. (**b**) Detection of chlorophyll fluorescence in WT and OE plants under different time points of salt stress. The images were take using an IMAGING-PAM chlorophyll fluorometer. (**c**) The SPAD value in WT and OE plants before and after salt stress treatment. (**d**) The Fv/Fm ratio in WT and OE plants before and after salt stress treatment. (**e**) The POD activity in WT and OE plants before and after salt stress treatment. (**f**–**h**) The content of leaf Na^+^, K^+^, and the ratio of Na^+^/K^+^ in WT and OE plants before and after salt stress treatment. Different letters (**a**–**f**) represent significant mean differences at *p* < 0.05.

Phylogenetic tree analysis indicated close homology between AvCIPK11 and AtCIPK11 proteins (Figure 1). Previously, the AtCIPK11 was identified as a positive regulator of stress responses [38]. To investigate the function of *AvCIPK11* in salt tolerance, an expression vector pBI121-AvCIPK11-GFP was transformed into Ac ‘Hongyang’ through the *Agrobacterium*-mediated transformation. The successful transformants were identified by using a specific pair of primers in the polymerase chain reaction (PCR) analysis (Figure 6). Based on higher expression levels, three positive lines for AvCIPK11 were chosen for further salt stress assays (Figure S2). The OE (overexpressed) and WT (wild type) plants were grown on Murashige and Skoog (MS) medium containing 1.0% (*w*/*v*) NaCl and plant samples were taken at 0 h (control), 24 h, and 7 days after salt stress treatment. The phenotype analysis showed that the OE plant performed better than WT (Figure 7a,b). Under salt stress, the SPAD and Fv/Fm levels in the OE plants were significantly higher than that of WT plants (Figure 7c,d). In addition, the POD (peroxidase) activity in OE plants was higher than that in WT plants (Figure 7e). In contrast, the levels of Na^+^ and Na^+^/K^+^ ratio in OE plants were lower than in the WT plants under salt stress (Figure 7f–h).

### 2.6. AvCIPK11 Activates the Expression of Salt Stress-Related Genes

**Figure 8:**
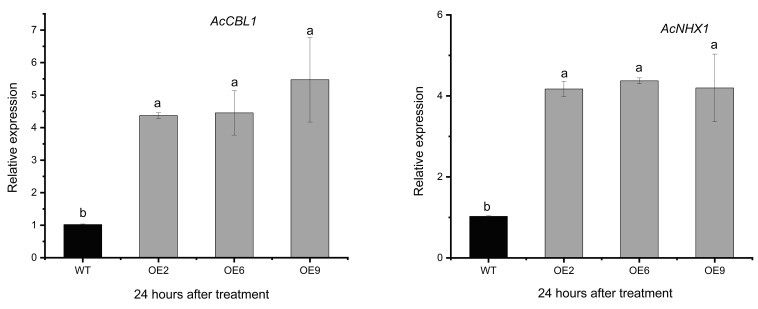
Expression analysis of *AcCBL1* and *AcNHX1* in OE plants. WT: ‘Hongyang’ kiwifruit; OE2, OE6, and OE9 repression the three transgene lines. Different letters (a–b) represent significant mean differences at *p*<0.05.

We carried out RT-qPCR analysis in OE plants to analyze the expression levels of salt stress-related genes. Previously, researchers reported that CIPK genes were regulated by *CBL* genes to influence plant adaptation to salt stress [12,13]. In the current study, expression levels of *AcCBL1* and *AcNHX1* were significantly higher in OE plants than in WT after 12 h of salt treatment (Figure 8). These results suggest that *AvCIPK11* in coordination with some salt-related genes (*AcCBL1* and *AcNHX1)* might enhance plants’ salt tolerance.

## 3. Discussion

The advent of sequencing technologies (RNA-seq etc.) opens up new horizons to unravel the complex molecular mechanisms of cellular events [39]. However, it is a daunting task to identify gene families in a non-sequenced organism. Salt stress imposes the most devastating effect on plants. Ion transport (especially Na^+^/K^+^ homeostasis) is one of the major methods to reduce the harm of salt stress. The CIPK gene family, an important gene family that plays a key role in regulating abiotic stress tolerance in plants, has been functionally characterized for biotic and abiotic stress tolerance in plants [8,40,41,42]. Many *CIPK* genes have been reported to regulate ion homeostasis under salt stress [43]. However, the unavailability of reports on expression patterns and the function of *CIPKs* from *Actinidia valvata* under salt stress compelled us to conduct the current study. Based on transcriptome data, we identified 42 *CIPK* genes in *A. valvata* (ZMH). According to the phylogenetic analysis, we classified them into four different groups based on their homology with *Arabidopsis CIPKs*. The presence of specific motifs in various phylogenetic groups indicated the functional orientation of members of these groups. *AvCIPK11* is a homologous gene to *AtCIPK11*. It was found that *AtCIPK11* enhanced cadmium stress tolerance and decreased drought stress tolerance in plants [38,44]. In addition, the overexpression of *NtCIPK11*, a homologous gene to *AtCIPK11*, promoted salt and drought tolerance in *Arabidopsis* [45]. The CIPK, a Ca^2+^ signal transductor, may play a key role in stress tolerance. The overexpression of *AvCIPK11* enhances kiwifruit salt stress. Our findings shed light on the molecular mechanism by which plants cope with salt stress by modulating ion homeostasis and ROS scavenging. 

Under salt stress, plans transduce signals through four different pathways, including electromagnetic signaling, calcium signaling, ROS signaling, and osmotic signaling [46]. The dehydration caused by salt stress activates the ion channels in plant cells. The calcium (Ca^2+^) channels and CIPKs regulate the hyperosmotic tension within plant cells under salt stress to protect them from death. The CIPK family members are the main ion signal transductor in plants. The rapid accumulation of calcium ions (Ca^2+^) in cytosol helps plant cells to sense and respond to salt stress. It is speculated that an initial overaccumulation of Ca^2+^ ions upon salt stress occurs due to *CIPKs, CBLs, CaM, CMLs,* and *CDPKs* [47]. The CIPK family members are the main ion signal transductors. All the *AvCIPK* genes in our experiment have the conserved domain (Figure 3). The presence of conserved domains (CIPK_C, STKc_SnRK3, and PKc_like superfamily) in AvCIPK proteins ensured their functional integrity. The AvCIPK genes were divided into four groups, unlike CIPKs in *A. thaliana* and rice, which were divided into five groups [30,48]. These results might occur due to species differences (Figure 1). The gene structure and evolution analysis presented new insights into the functional annotation of *AvCIPK* genes. The most interesting findings were that the CIPKs gene clustered into the same phylogenetic groups in different species had the same function [30]. The *AvCIPK* genes which did not cluster into AtCIPK phylogenetic groups might attribute to functional specificity. The conserved motif analysis showed that the CIPK family member had the common motif composition of motif 2, motif 3, motif 11, and motif 14, which corresponded the findings in tomato [49]. Motif 7 and motif 8 were identified within the NAF domain and PPI domain [32]. Most AvCIPK proteins have been demonstrated to contain both domains, suggesting the potential function of *AvCIPKs* in participating salt stress.

In the past, many CIPK genes were reported to enhance the salt stress tolerance of apple, cotton, soybean, and *Nitraria tangutorum* [45,50,51,52]. The *MdCIPK13* gene was induced by salt stress in apple [50]. Moreover, antioxidant metabolites were increased in the overexpression of *MdSOS2L1* in apple and tomato [53]. The induction of *ZmCIPK24* expression in maize indicated the positive function of this regulator in salt stress [40]. We analyzed all *AvCIPKs* that had consistently high expression after 72 h of salt stress and identified five candidate genes (*AvCIPK6*, *AvCIPK11*, *AvCIPK12*, *AvCIPK16*, and *AvCIPK35*), which were significantly up-regulated by salt. We abandoned the low quality proteins AvCIPK12 and AvCIPK35 based on functional annotation. The SOS3-SOS2-SOS1 module was identified as a typical pathway to regulated ion homeostasis. The AvCIPK6 and AvCIPK11 were annotated as SOS3-interacting proteins based on KEGG annotation. The *AvCIPK11* shared high homology with *AtCIPK11* and a high expression level under salt treatment and was screened for further function research. The result of subcellular localization of AvCIPK11 protein indicated it as a signal transporter for salt stress, which was consistent with the findings in other CIPK family members in *Arabidopsis* [54]. The *AvCIPK11* is an important signal transporter in kiwifruit that plays a role in ion transduction under salt stress. 

We performed the transformation of *AvCIPK11* in kiwifruit to determine its function under salt stress. The overaccumulation of Na^+^ ions under salt stress causes serious damage to plant cells. Chlorophyll concentration is a key indicator of leaves’ health. Previous research found that soil salinity stress interrupted the enzymatic activities of chlorophyll biosynthesis [55]. The overexpression of *TaCIPK14* in wheat enhanced chlorophyll content and decreased Na^+^ concentration [56]. Our findings are consistent with a previous study in which Fv/Fm ratio and SPAD value in the transgenic plant was higher than in WT plants (Figure 7c,d). The result showed that the leaves of WT kiwifruit were more vulnerable to salt stress damage than transgene kiwifruit leaves. Salt stress was followed by ionic stress, osmotic stress, and secondary oxidative stress that severely injured plants. The salt-tolerant wheat varieties had a lower Na^+^/K^+^ ratio and reduced oxidative stress than salt-sensitive varieties [57]. Excessive Na^+^ ion accumulation in cells interfered with K^+^ homeostasis, resulting in cell metabolic harm [58]. The activity of peroxidase (POD) was also influenced by CIPK family members in plants [24]. The molecular regulatory mechanism of CIPK family members during salt stress differs across plant species. In *Arabidopsis*, the CBL-CIPK module has been shown to play a vital role in regulating Na^+^, K^+^, and NO_3_^−^ between cells [15,59,60,61]. Therefore, maintaining a balanced K^+^/Na^+^ ratio has emerged as a crucial factor for the regulation of salt stress in plants [62,63]. In the present study, the overexpression of *AvCIPK11* decreased the Na^+^/K^+^ ratio in transgene kiwifruit plant leaves (Figure 7f–h). The salt-induced reactive oxygen species in plants are highly harmful to plant growth and development and ROS scavenging is essential for plants to maintain their viability under salt stress [64]. Plants produce many antioxidant enzymes (POD, SOD, CAT) to protect them from oxidative damage. The higher level of POD safeguarded kiwifruit OE plants from oxidative stress. In addition, POD activity was maintained at a high level, regardless of salt stress. A possible explanation for this might be that the overexpression of *AvCIPK11* triggered the expression of POD-related genes and enhanced the ability of oxidase scavenging in plants.

The CBL-CIPK complex is involved in Ca^2+^ signaling induced by abiotic stress. The CIPK genes often interact with CBL, which is a serine/threonine protein kinase [60,65]. The wheat TaCBL-TaCIPK24 signaling pathway plays a key role in salt tolerance, and overexpression of *TaCIPK24* in *Arabidopsis* increased the Na^+^ efflux and enhanced the ROS scavenging ability [66]. In *Arabidopsis*, the CBL10-CIPK8-SOS1 protein complex co-regulated Na^+^ efflux and enhanced salt tolerance [43]. In *Hordeum brevisubulatum*, the HbCBL4/10-HbCIPK2 complex modulated *HbSOS1L* to exclude Na^+^ [67]. The Na^+^/H^+^ antiporters (NHXs) excreted excess cytosolic Na^+^ into the vacuole [68]. Furthermore, the plasma membrane-located *NHX7* and *NHX8* (also known as SOS1) played a role in ion homeostasis [69]. The function of *NHXs* has been verified in *Arabidopsis* [70], *Beta vulgaris* L. [71], and rice [72] under salt stress. It is possible to hypothesize that these conditions are likely to occur in kiwifruit. Therefore, we measured the expression level of *AcCBL* and *AcNHX* genes in OE kiwifruit plants. The expression level of *AcCBL* and *AcNHX* was highly induced in OE plants and the (Figure 8). These results were in agreement with those obtained in soybean [73]. The *AvCIPK11* might enhance plant salt tolerance by regulating ion homeostasis-related genes. Hence, we can infer that *AvCIPK11* can increase salt tolerance by improving ROS scavenging and maintaining ion homeostasis. Further research should be undertaken to investigate how *AcCBL1* and *AcNHX1* coordinate with *AvCIPK11* to co-regulate plant salt tolerance. 

## 4. Materials and Methods

### 4.1. Identification of CIPK Family Members in A. valata and Arabidopsis 

We obtained the CDS, protein, and the full-length gene sequences of kiwifruit CIPKs from our previously published data [37]. Twenty-five AtCIPK protein sequences were retrieved from The Arabidopsis Information Resources database (TAIR, https://www.Arabidopsis.org/, accessed on 6 May 2022) and blasted to *A. valvata* transcriptome to obtain AvCIPK protein sequences. Each member of the AvCIPK protein was subjected to InterProscan (http://www.ebi.ac.uk/Tools/pfa/iprscan/, accessed on 10 May 2022) and ScanProsite (https://prosite.expasy.org/prosite.html, accessed on 10 May 2022) database to confirm the presence of conserved domains protein kinase (PF00069) and the NAF (PF03822). The conserved domains were detected by NCBI Conserved Domains (https://www.ncbi.nlm.nih.gov/cdd/?term=, accessed on 11 May 2022). The sequences harboring both conserved domains were identified as candidate sequences. All the sequences for CIPK proteins are shown in Supplementary Table S2. 

### 4.2. Phylogenetic Analysis and Conserved Motif Identification

The multiple sequence alignment analyses of 42 AvCIPKs and 25 AtCIPKs were carried out using the Clustal W method with a default settings, and then a phylogenetic tree was constructed by using MEGA-X (Mega Limited, Auckland, New Zealand) with the neighbor-joining (NJ) method and 1000 bootstrap replicates [74]. The phylogenetic tree was visualized by the interactive tree of life (iTOL) (https://itol.embl.de/itol.cgi, accessed on 12 May 2022). The conserved motifs in AvCIPKs were analyzed by the Multiple Em for Motif Elicitation suit (MEME; http://memesuite.org/tools/meme, accessed on 13 May 2022). A total of 15 conserved motifs were identified in each of the AvCIPK proteins. The motif with an e-value of less than 1×10^−60^ was screened for further study. The model of fragments per kilobase of exon per million (FPKM) from RNA-seq data was used to assess the expression levels of *AvCIPK* under salt stress. The heatmap of FPKM values of *AvCIPK* was constructed by TBtools (https://github.com/CJ-Chen/TBtools/releases, accessed on 2 June 2022) [75].

### 4.3. Plant Materials Preparation and Application of Salt Treatment

The salt-tolerant genotype ZMH was grown in a greenhouse at Zhengzhou Fruit Research Institute, Chinese Academy of Agriculture Science, Henan Province, China (Latitude 34°43′N, Longitude 113°39′E, and Altitude 111 m). At the 4–6 leaf stage, kiwifruit plants were treated with 0.4% NaCl per net weight of the growing medium in the pot. All the treatments were repeated thrice and there were five plants in each replication. We took samples from the roots of kiwifruit plants at 0 h, 12 h, 24 h, and 72 h after salt treatment. All samples were immediately frozen in liquid nitrogen and stored at −80 °C for subsequent use.

### 4.4. RNA Isolation and RT-qPCR Analysis

Total RNA was extracted by using the Plant Total RNA purification kit (Waryoung, Beijing, China). Genomic DNA was removed by DNase I (Thermos Scientific, Waltham, MA, USA) from the total RNA. Then, the cDNA was synthesized using the First Strand cDNA Synthesis Kit (TOYOBO, Tokyo, Japan). The pair of primers for RT-qPCR was designed by Primer Premier 5.0 (reference). The RT-qPCR reaction was performed by a previously described method [76]. The gene expression levels were calculated using the 2^−∆∆Ct^ method [77]. The pair of primers used in this study is listed in Table S4.

### 4.5. Subcellular Localization Analysis

The full-length coding sequence (CDS) of *AvCIPK11* without a stop codon was cloned into the vector pBWAVHS-GLosgfp with N-terminal fusion of AvCIPK11 to the GFP report gene. The recombinant vector pBWAVHS-AvCIPK11-GLosgfp and empty vector pBWAVHS-GLosgfp were transformed into tobacco leaves (*Nicotiana benthamiana*) by *Agrobacterium-*mediated transformation. Then, a laser scanning confocal microscope (Nikon C2-ER, Tokyo, Japan) was used for detecting the GFP signal in transformed leaves. For GFP detection, excitation was carried out at 488 nm wavelength and detection at 510 nm. For chloroplast fluorescence detection, the excitation wavelength was 640nm and the detection wavelength was 675 nm. All the pairs of primers used in this analysis were shown in Table S4.

### 4.6. Construction of Overexpression Vector, Transformation, and Regeneration of Transgenic Kiwifruit Plants

The full-length ORF of AvCIPK11 was cloned from root samples of ZMH by using specific pair of primers (Table S4). Afterward, the ORF of AvCIPK11 without a stop codon was inserted into the PBI121-GFP vector driven by the CaMV35S promoter and the recombinant plasmid was transformed into leaf discs of *A. chinensis* (a salt sensitive cultivar ‘Hongyang’) according to a previously described method [78]. The transgenic plants were obtained after six months and were later propagated on an MS medium [79]. We identified three positive transgenic lines via PCR analysis and fluorescence imaging. The pair of primers used for this study is presented in Table S4.

### 4.7. Salt Tolerance Assay

To evaluate the salt tolerance ability of transgenic kiwifruit plants, OE and WT plants were grown on an MS medium containing 1.0% (*w/v*) NaCl, and samples were taken at 0 h, 24 h, and 7 d. After 30 min of dark treatment, an IMAGING-PAM chorophyll fluorometer (Walz, Effeltrich, Germany) was used to capture chlorophyll fluorescence images. The maximum quantum efficiency of photosystem II (Fv/Fm) was measured by Imaging WinGegE software (Walz, Effeltrich, Germany) [80]. The chlorophyll contents were measured by SPAD meter (SPAD 502 plus, Minolta, Japan) [81]. The Na^+^ and K^+^ ions were determined according to the protocol of commercial kits (Nanjing Jiancheng Bioengineering Institute, Nanjing, China). The activity of POD was detected using a commercial kit (Solarbio, Beijing, China).

### 4.8. Statistical Analysis

All the statistical analyses were performed using SPSS 21.0 software (IBM Corporation, Chicago, IL, USA). The data were analyzed for significant differences by one-way ANOVA, and mean differences between the samples were analyzed by Tukey’s multiple comparisons test. The data samples were considered significantly different at *p* < 0.05. All the results were presented as the means ± SE (standard error).

## Data Availability

Sequence data from this work can be found in the NCBI database (SRA data).

## Supplementary Materials

The following supporting information can be downloaded at: https://www.mdpi.com/article/10.3390/ijms24010805/s1.
