# Supplementary material for: Transcriptome-Wide Identification and Functional Characterization of CIPK Gene Family Members in Actinidia valvata under Salt Stress
Source: Int J Mol Sci. 2023 Jan 2;24(1):805. doi: 10.3390/ijms24010805 (PMC9821023; doi:10.3390/ijms24010805)
Supplement: Supplementary file 1 [file ijms-24-00805-s001.zip › supplymentary figs.pdf]

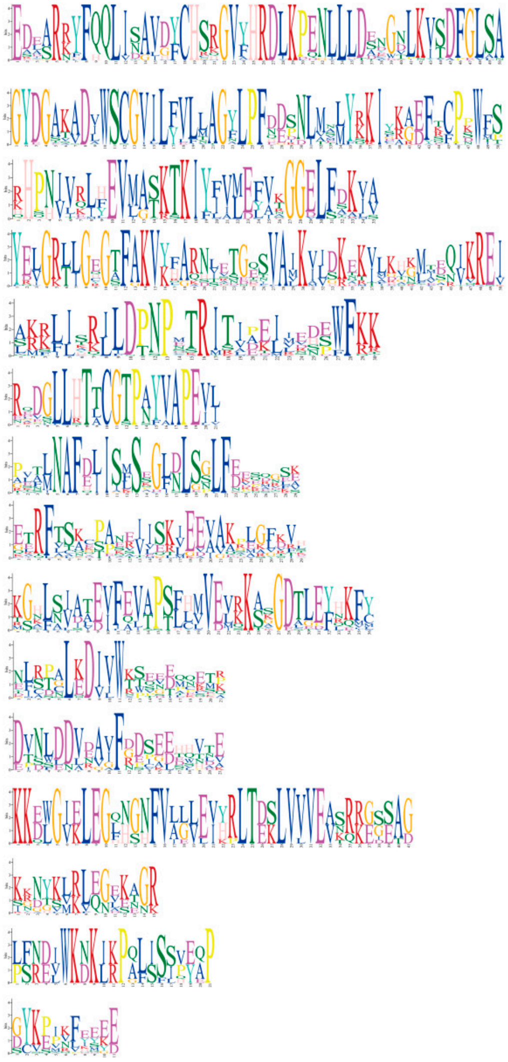

Motif	E-value	Site	Width
Motif 1	3.8×e^{-1696}	42	50
Motif 2	3.1×e^{-1671}	42	50
Motif 3	7.3×e^{-990}	41	35
Motif 4	2.0×e^{-1281}	42	50
Motif 5	8.6×e^{-741}	42	30
Motif 6	4.9×e^{-639}	40	21
Motif 7	1.5×e^{-614}	42	29
Motif 8	2.3×e^{-440}	41	29
Motif 9	1.1×e^{-568}	29	36
Motif 10	6.6×e^{-185}	25	21
Motif 11	1.1×e^{-184}	25	21
Motif 12	5.5×e^{-143}	9	41
Motif 13	4.1×e^{-114}	25	15
Motif 14	2.9×e^{-067}	9	21
Motif 15	6.0×e^{-060}	25	11

Figure S1. The conserved motif logos of amino acid sequences from AvCIPK proteins. A total of 15 logos were identified in kiwifruit CIPKs.The height of each stack indicates the conservation of the sequence at the position of the marker, and the height of each letter in each stack indicates the relative frequency of the corresponding amino acid.

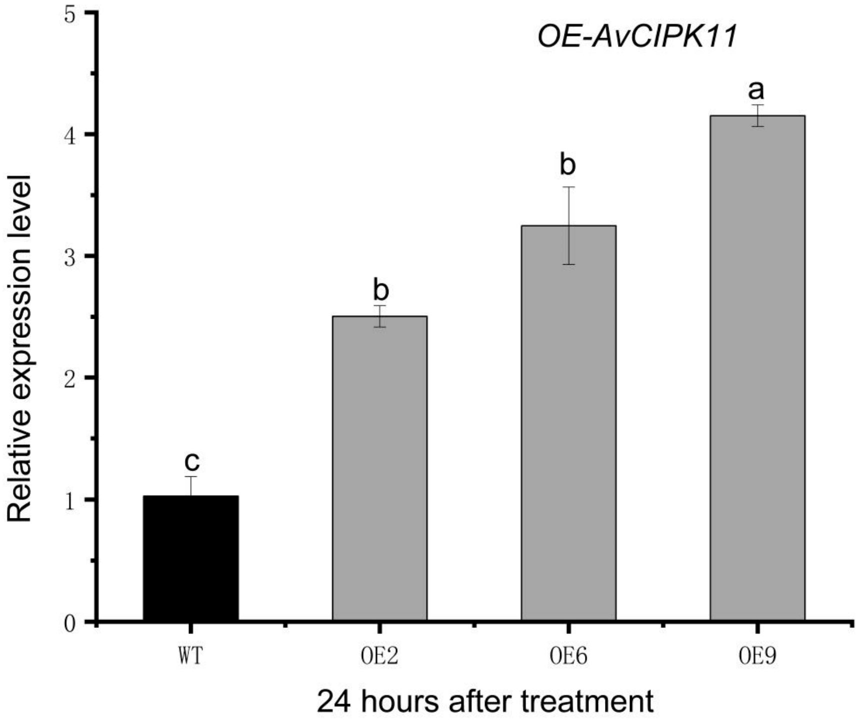

Figure S2. The expression analysis of OE-*AvCIPK11* in transgenic kiwifruit plants.
